# Supplementary figures and images for: Shoe configuration effects on equine forelimb gait kinetics at a walk
Source: PeerJ. 2025 Feb 26;13:e18940. doi: 10.7717/peerj.18940 (PMC11871903; doi:10.7717/peerj.18940)

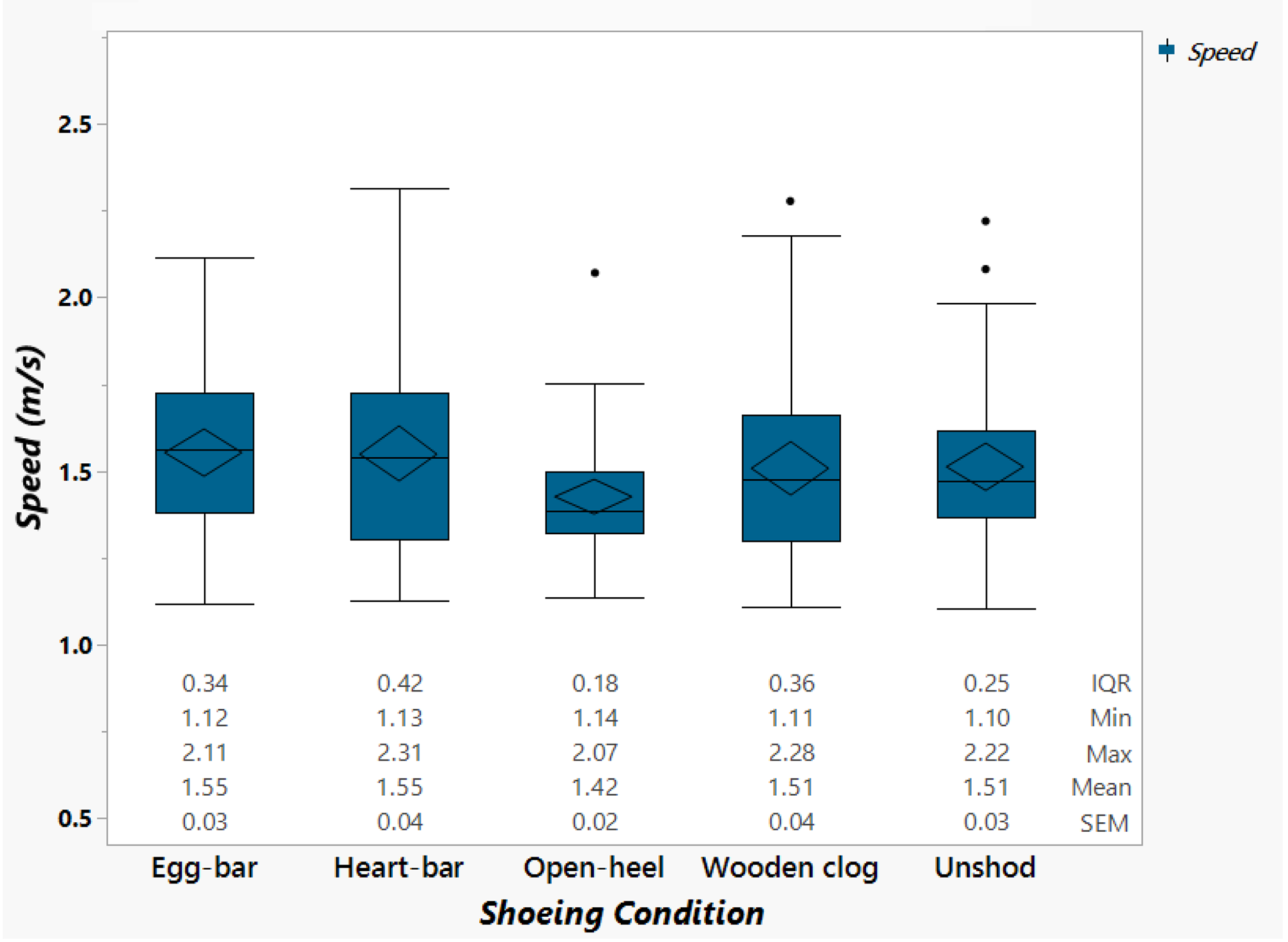

Supplement: Supplemental Information 1 — Box plots illustrating speed of gait trials with horses (n = 6) shod with egg-bar, heart-bar, open heel, or wooden clog shoes and while unshod. The values below each data set are the interquartile range (IQR), minimum value (Min), maximum value (Max), mean (Mean) and standard error of the mean (SEM). [file peerj-13-18940-s001.png]

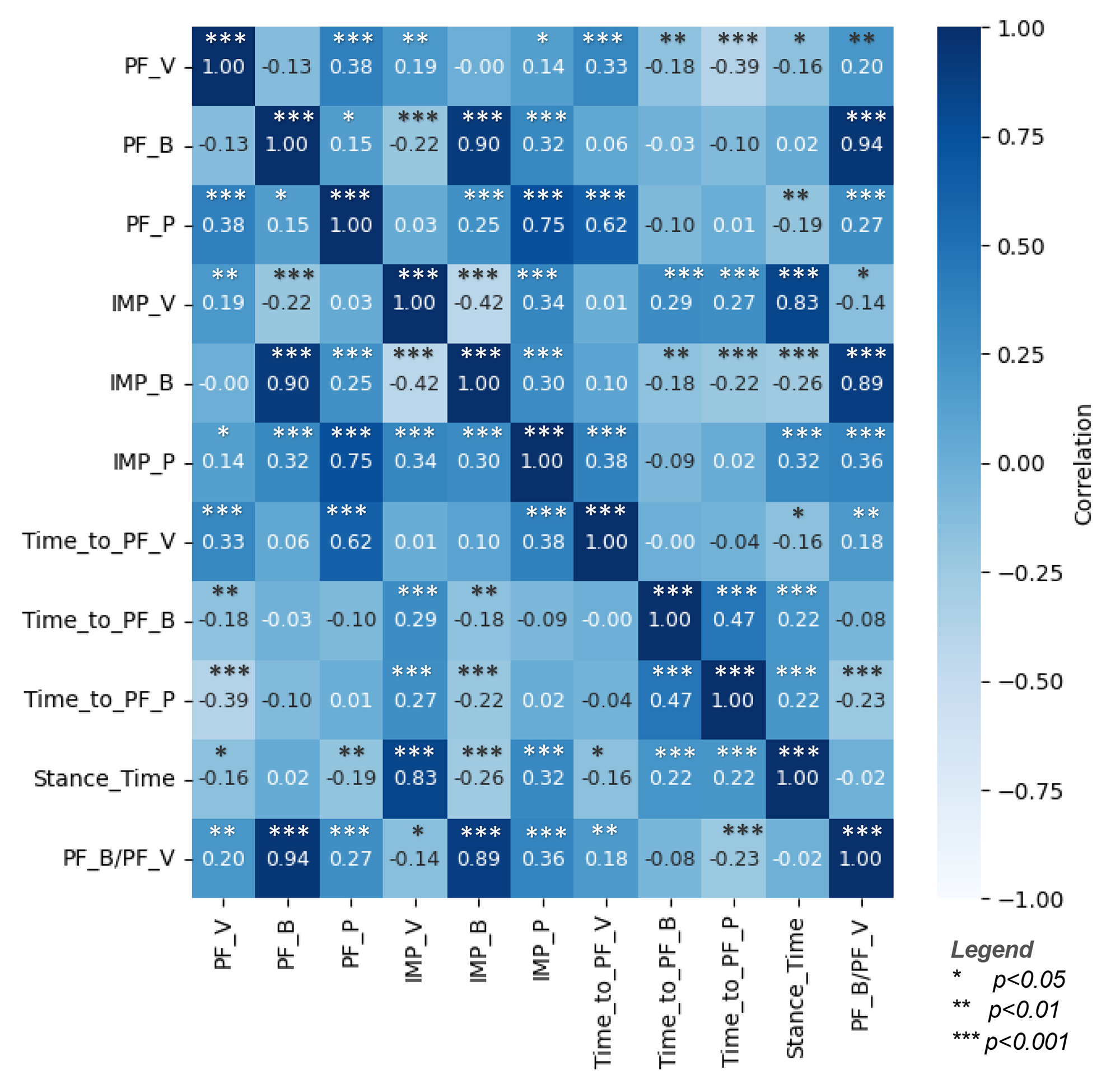

Supplement: Supplemental Information 2 — Correlation coefficients between equine forelimb kinetic gait variables recorded from non-lame horses (n = 6) at a walk. The scale on the right side of the matrix shows the colors on the graph that represent the correlation coefficients among the variables ranging from −1 (dark blue), through 0 (light blue), to +1 (off-white). Asterisks above each correlation coefficient show the Pearson’s test for correlation p-value (∗p-value < 0.05, ∗∗p-value < 0.01, ∗∗∗p-value < 0.001). [file peerj-13-18940-s002.png]
